# An Overview on the Application of Chemometrics Tools in Food Authenticity and Traceability

**DOI:** 10.3390/foods11233940

**Published:** 2022-12-06

**Authors:** Raúl González-Domínguez, Ana Sayago, Ángeles Fernández-Recamales

**Affiliations:** 1 Instituto de Investigación e Innovación Biomédica de Cádiz (INiBICA), Hospital Universitario Puerta del Mar, Universidad de Cádiz, 11009 Cádiz, Spain; 2 Agrifood Laboratory, Faculty of Experimental Sciences, University of Huelva, 21007 Huelva, Spain; 3 International Campus of Excellence CeiA3, University of Huelva, 21007 Huelva, Spain

**Keywords:** chemometrics, food authenticity, food traceability, multivariate analysis

## Abstract

The use of advanced chemometrics tools in food authenticity research is crucial for managing the huge amount of data that is generated by applying state-of-the-art analytical methods such as chromatographic, spectroscopic, and non-targeted fingerprinting approaches. Thus, this review article provides description, classification, and comparison of the most important statistical techniques that are commonly employed in food authentication and traceability, including methods for exploratory data analysis, discrimination, and classification, as well as for regression and prediction. This literature revision is not intended to be exhaustive, but rather to provide a general overview to non-expert readers in the use of chemometrics in food science. Overall, the available literature suggests that the selection of the most appropriate statistical technique is dependent on the characteristics of the data matrix, but combining complementary tools is usually needed for properly handling data complexity. In that way, chemometrics has become a powerful ally in facilitating the detection of frauds and ensuring the authenticity and traceability of foods.

## 1. Introduction

The authenticity of foods and beverages is an issue of utmost importance for all actors participating in the food chain [1]. Whereas producers must ensure the quality and added value of their products, food authorities are responsible for controlling the safety and traceability of the foods and beverages that can be found in the market. The awareness of consumers has also significantly increased over recent years, demanding safe, honest, and properly labeled foods as well as the promotion of local/organic products and the implementation of environmentally friendly and animal welfare-conscious production methods. Nevertheless, this market niche of healthier, safer, autochthonous, and environmentally friendly foods, which consequently are more expensive, is highly susceptible to adulterations and frauds. In this vein, food authentication encompasses many different issues revolving around adulteration, mislabeling, and misleading statements about origin (e.g., geographical origin, cultivar/breed), production method (e.g., organic/conventional production) or processing techniques (e.g., conservation, stabilization) [2]. In particular, traceability issues regarding geographical origin are one of the most important food authenticity problems in Europe, especially considering that the European quality policy has strict regulations to recognize and protect the names of products related to certain locations and/or to specific processing methods [3]. Among the geographical indications, the Protected Designation of Origin (PDO) indicates the area where food products were produced, whereas the Protected Geographical Indication (PGI) recognize territories where at least one of the three manufacturing processes (production, transformation, or elaboration) occurred. On the other hand, the Traditional Specialties Guaranteed (TSG) indication protects traditional production methods. Accordingly, the implementation of reliable, cost-effective, and powerful analytical methods has emerged as an urgent need in the food industry to ensure the authenticity and traceability of food products and, consequently, to evaluate their organoleptic, nutritive, and nutraceutical properties [4,5].

The large and complex datasets that state-of-the-art analytical methods usually generate make the implementation of advanced chemometric techniques, aimed at extracting as much valuable information as possible from the raw data, mandatory. Thus, chemometrics refers to the application of statistical and mathematical tools for identifying the underlying relationships between variables. This enables the construction of statistical models able to interpret the characteristics of the system on the basis of available chemical data, which can subsequently be employed for classification, discrimination, and prediction purposes. In this review article, we provide a description, classification, and comparison of the most commonly employed chemometric techniques in food authentication and traceability. Furthermore, we also discuss some relevant issues that are sometimes underestimated in this field, such as the importance of data preprocessing and further validation of the models obtained.

## 2. Analytical Approaches in Food Authenticity Research

Two complementary analytical strategies are available nowadays for food authentication and traceability, namely targeted and non-targeted approaches [6,7]. Targeted methods are based on the analysis of the components that are expected to be present in the sample, whereas the non-targeted approach relies on comprehensive chemical analysis with the aim of simultaneously detecting as many substances as possible [7,8]. Among omics-based methods, metabolomics is one of the most potent techniques for food authentication purposes, since it provides holistic information about the global metabolite composition derived from metabolism and interactions with the environment [9]. In this respect, chromatographic, spectroscopic, spectrometric, and molecular biology methods are currently the most widely employed techniques to investigate the authenticity of food products (Table 1).

Chromatography coupled with spectroscopic or spectrometric detection has been proven to be a suitable tool for determining and quantifying individual components or compound classes in food samples [10]. Liquid chromatography coupled with ultraviolet-photodiode array detection (DAD) or mass spectrometry (MS) is routinely employed for the analysis of a wide range of analytes because of its precision, low cost, and versatility [11,12,13]. In this respect, the boom in ultra-high-performance liquid chromatography (UHPLC) has further improved its resolution and sensibility performance, thereby reducing the time needed for accomplishing qualitative and quantitative analyses and consequently enabling the simultaneous determination of different compound classes in a single run [14]. On the other hand, gas chromatography is frequently applied for profiling volatile substances (e.g., aroma-related compounds, lipid fractions) [15,16,17]. 

As minerals are mainly absorbed by plants and animals through soil and water resources, multi-elemental techniques have demonstrated great utility for assessing geographical origin, environmental conditions of the growing/breeding area, and crop management [18,19]. Furthermore, the measurement of isotopic ratios has also been proposed as a candidate authenticity marker [20]. For instance, oxygen-18 (δ^18^O) and deuterium (δ^2^H) are strongly impacted by hydrological factors, so they can serve to differentiate food samples according to their origin. The carbon isotopic ratio (^13^C/^12^C) is affected by climatic conditions, plant type, and agricultural practices. Finally, nitrogen isotopic analysis (δ^15^N) allows the differentiation of conventional from organic farming because the nitrogen isotope content in synthetic fertilizers is much lower than in organic ones [21]. 

Regarding non-targeted fingerprinting, it should be noted that various analytical platforms have been proposed over recent years for analyzing complex metabolomes. However, as each one of these techniques has their own advantages and limitations, the combination of various approaches is nowadays the common practice for performing comprehensive analysis [22]. The use of MS-based platforms is nowadays widespread because of their ability to identify and (semi)quantitate a multitude of compounds with high sensitivity and high-throughput capabilities. Although high-resolution mass spectrometry is the gold standard for non-targeted metabolomics, the implementation of large-scale targeted platforms has emerged in recent years for comprehensive and quantitative metabolomics fingerprinting [23]. Alternatively, spectroscopic techniques, such as near-infrared spectroscopy (NIR), mid-infrared spectroscopy (MIRS), RAMAN spectroscopy, ultraviolet-visible (UV-Vis) spectroscopy, and nuclear magnetic resonance (NMR), have also demonstrated great potential for food fingerprinting due to their capacity to simultaneously detect many compounds in a fast, non-destructive, and cost-moderate manner [24,25,26].

Finally, a variety of molecular biology approaches are also well-established for the analysis of nucleic acids, proteins, and peptides as markers of adulteration and authenticity. In particular, as the DNA sequence is unique to each living organism, DNA-based methods have traditionally been employed to track raw materials across the whole industry process. These molecular techniques range from traditional molecular marker-based methods (e.g., single nucleotide polymorphisms) to more recent single region approaches (e.g., DNA barcoding, isothermal amplification-based methods) and next-generation sequencing-based methods (e.g., DNA metabarcoding) [27]. However, although these tools have been demonstrated to be highly specific and sensitive, they are largely qualitative in nature. Accordingly, great efforts have been made recently to develop quantitative PCR-based and proteomics assays [28].

## 3. Chemometric Approaches in Food Authenticity Research

**Figure 1:**
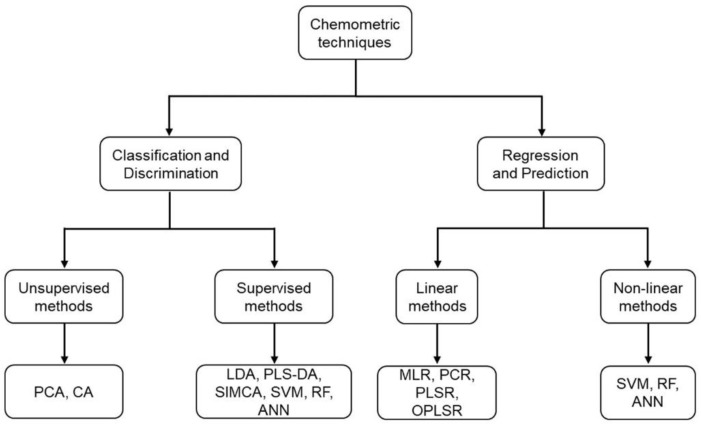
Classification of the chemometric techniques commonly used in food science.

As a result of the large amount of data that the abovementioned analytical techniques generate, the use of advanced multivariate chemometric tools is mandatory for extracting as much valuable information as possible (Figure 1). Multivariate statistical analysis can be performed either by using unsupervised or supervised methods. Unsupervised analysis is based on the identification of sample interrelationships without prior information about class membership [29], whereas supervised methods use a training stage, in which samples are assigned to known groups to build mathematical models [30]. In turn, the most common multivariate methods that are used in food authentication research can be divided into three classes: (i) exploratory data analysis; (ii) discrimination and classification; and (iii) regression and prediction.

### 3.1. Data Preprocessing

Prior to performing any statistical analysis, the application of preprocessing tools is usually mandatory for enhancing the quality of the data matrix via removing unwanted variability sources (e.g., analytical variation, experimental artifacts). This is typically accomplished by applying various sequential steps, including data cleaning (i.e., imputation of missing values, removal of outliers), scaling, and transformation [31]. Missing values are normally handled in two complementary ways. First, variables with a proportion of missing values above a certain cutoff (e.g., 20%) are discarded and the remaining ones are then imputed, for which different methods are available. The simplest imputation methods rely on substitution (e.g., mean, median, minimum value, limit of detection), but other multivariate methods have been proposed over recent years (k-nearest neighbors, kNN). On the other hand, outliers are a serious source of distortion in statistical results, which makes the implementation of powerful techniques for their proper detection and removal necessary. For this purpose, some statistical methods can be employed to quantify the boundary and amount of data outliers (e.g., Euclidean distances). However, when the number of variables under investigation is large, other multivariate approaches (e.g., principal component analysis, clustering analysis) represent a suitable strategy to easily detect outlier samples. Then, data scaling is often needed when the input variables have different scales (e.g., wide range of concentrations). Thus, the aim of this step is to use a scaling factor to adjust for the dissimilarities in fold differences between the different analytes. To this end, the scaling factor can be a measure of the data dispersion (e.g., the standard deviation) or a size measure (e.g., the mean). Finally, different transformation algorithms are usually applied to correct heteroscedasticity issues, to convert multiplicative relations into additive relations, and to increase the symmetry of skewed distributions.

### 3.2. Exploratory Data Analysis

Exploratory data analysis (EDA) is normally employed as a first step to visualize the main characteristics of the dataset. For this purpose, unsupervised pattern recognition techniques are normally applied to reduce the dimensionality of complex data matrices without losing information. This enables projecting samples into a low-dimensional space (e.g., scatter plots, dendrograms) with the aim of inspecting sample groupings, detect outliers, etc.

#### 3.2.1. Principal Component Analysis

Principal component analysis (PCA) is a powerful and commonly used tool for reducing the dimensionality of vast sets of data [32]. Accordingly, the most important application of PCA is compressing the n-dimensional data structure into a lower number of components, normally fewer than three, which are named principal components (PCs). The PC is a new variable describing the samples (objects), which is calculated as a linear combination of the primal ones. The selection of the first PC is carried out in such a way that it retains as much information as possible. The rest of the PCs are chosen to be orthogonal to the previous ones and to explain as much as possible the variation left unexplained before. The eigenvalues that are generated during PCA indicate the extent of information that is explained by each of the computed PCs. The most widely implemented criteria for selecting the optimum number of PCs is to solely preserve those combinations with eigenvalues above 1 (i.e., Kaiser criterion). The projections of the objects in the plane defined by the computed components are named scores plots, which are weighted sums of the original variables. The magnitude and sign of these weights, or loadings (coefficients of the combination linear of original variables), are indicative of their importance in explaining the data’s complexity.

#### 3.2.2. Cluster Analysis

Cluster analysis (CA) enables the grouping of similar observations into a number of clusters based on the observed values of several variables for each individual. These subclasses may reveal patterns related to the phenomenon under study. To conduct CA, similarity measures must be first computed between observations and between clusters once observations begin to be grouped into clusters. The clustering procedure is usually based on Euclidean distance (d), although other metrics may also be adopted to measure distances between objects, such as the squared Euclidean distance, the Manhattan distance, the Pearson coefficient of correlation, or user-defined distances. Clustering methods can in turn be categorized into four subtypes: hierarchical, optimizing, density-seeking, and clumping techniques. In hierarchical methods, classes are subclassified into groups by repeating the modeling in iterative steps to yield a tree, where study groups are represented as branches. Optimizing techniques are based on the construction of clusters by selecting the optimal grouping criteria. The obtained classes must be mutually exclusive, where the observations are plainly divided in different sets. In density-seeking (also called mode-seeking clustering), the clusters are detected and generated by searching areas on a graphical representation containing concentrations of data measures. Finally, clumping methods use a modification of the density-seeking approach based on weighting specific variables to assign the clusters in a manner where obtained groups can be overlaid on graphic projections. Among them, the most common form of analysis is the hierarchical cluster analysis (HCA) tool [32], for which two distinct algorithms can be applied, agglomerative (grouping observations) or divisive (dividing the data set), the former being the most widely used in practice. In the agglomerative case, different linkage functions can be employed, including single, average, complete, and Ward’s linkage, which may yield distinct clusters with specific properties. The outcome from HCA is normally represented as clustering dendrograms or heat maps.

### 3.3. Discrimination and Classification

Discrimination and classification methods are employed for predicting an object’s belonging to a given category based on its characteristics [33]. For this purpose, the model is first built using a training set, which must be composed of a set of objects whose categories and values for the predictor variables are known a priori. The assignment of objects to categories must be exhaustive (i.e., every observation is included in one category) and mutually exclusive (i.e., there is no observation belonging to various categories). Then, the model is applied to predict the category of new objects. The aim of discriminant analysis techniques, also known as ‘hard modelling’, is to discriminate between classes based on splitting the data dimensionality in different regions, each one corresponding to a class. On the other hand, class-modelling analysis, or ‘soft modelling’, is based on creating a separate model for every category and identifying a defined area of the data space for each class (i.e., class space).

In this respect, it should be noted that supervised methods that are normally employed for classification and discrimination purposes are prone to overfitting, especially when the number of variables exceeds the number of observations, which makes the implementation of validation tools mandatory [29]. External validation is the most powerful approach, but it is only applicable when the sample size is large enough to be split into separate and independent training, calibration, and test sets. When a validation set is not available, cross-validation is a reliable strategy for checking model performance. To conduct a cross-validation, the dataset must be divided into two groups containing the same percentage of samples from each class, a training set for model construction and a test set for goodness assessment. This procedure is repeated several times to avoid conclusions by chance, in which samples are randomly split to ensure that they all have the same probability of belonging to the training set. Thereby, the model performance can be evaluated by computing the percentage of correctly classified samples within the training (i.e., recognition capacity) and validation (i.e., prediction capacity) sets. A third validation approach consists in calculating the sensitivity and specificity of the models using the confusion matrix. Here, sensitivity is computed as the percentage of cases belonging to a determinate class that are correctly classified, whereas the specificity refers to the percentage of cases not belonging to a class that are not classified in this class.

#### 3.3.1. Linear Discriminant Analysis

Linear discriminant analysis (LDA) is a supervised tool where a number of orthogonal discriminant functions (DF) equal to the number of categories minus one are generated. Discriminant functions are computed as linear combinations of the predictor variables and used to differentiate study groups by controlling the within-class and between-class ratios. The discriminant capacity of DFs can be assessed through the measurement of the Wilks’ lambda parameter, which is calculated for the global model after excluding the selected variable. Afterward, a forward stepwise algorithm is applied to retain the functions that must be considered in the final model. Accordingly, the assessment F values are regarded as suitable criteria for including or removing predictors in LDA. Furthermore, Wilks’ lambda and F values can be employed for testing the importance of each variable in the model. Once the DFs are computed, the groups can be differentiated by hyperplanes contained inside the space defined by DFs, and samples can be classified depending on their belonging to these subspaces (classification rule).

#### 3.3.2. Partial Least Square Discriminant Analysis

Partial least square discriminant analysis (PLS-DA) is a pattern recognition tool that relies on identifying an adequate number of latent variables/components with the aim of discriminating between previously established groups [34]. This technique is based on a classical PLS regression, which uses a category as the dependent variable to define class membership, of great utility in the case that the total of study variables is lower than the number of observations. The principle of PLS is finding the latent variables in the predictor matrix (X) that explain, as much as possible, the variability in the original data, and at the same time show strong association with the target value in the response matrix (Y). In other words, PLS-DA is intended to identify latent variables that enable simultaneously decomposing X and Y matrices, taking into consideration their covariance. The optimal number of components is usually assessed by cross-validation methods, so that the global performance of PLS-DA models is represented by the parameters R_Y_^2^, R_X_^2^, and Q^2^. R_Y_^2^ and R_X_^2^ are the proportion of Y and X variances explained by the model, respectively. Q^2^ is a measurement of the model prediction capacity. Furthermore, PLS models also enable obtaining an indicator of the discriminant ability of each variable by computing the variable importance for the projection (VIP) parameter. This is a weighted sum of squares of the PLS loadings that considers the proportion of Y-variance that is described by each component. Those variables showing VIP values larger than 1 can be regarded as the most discriminant ones, whereas values below 0.5 indicate little influence in the model.

#### 3.3.3. Soft Independent Modeling of Class Analogy

Soft independent modeling of class analogy (SIMCA) calculates significant PCs to classify samples based on their distance from the model representing each category. For this purpose, a training set is used to determine the number of PCs needed to describe the structure and boundaries of each class. Accordingly, objects are included within a certain class if they fall into the n-dimensional class-box limited by these boundaries. As each category is modeled independently of the rest, it is common to find overlapping between the spaces of each class. Therefore, three situations are possible: (1) the object falls within the boundaries of only one class, so it is unequivocally classified into that class; (2) the object is not located in any of the class boxes, so it is considered as an outlier; (3) the object falls in the overlapped region, so it is assigned as belonging to the overlapping classes (Massart). The classification results can be assessed by Coomans’ plots, a graphical tool for visualizing pairwise groupings, in which the axes depict the normalized orthogonal distance of the objects with respect to each individual model.

#### 3.3.4. K-Nearest Neighbors

K-nearest neighbors (kNN) is a simple method that does not require any distribution assumption and can be used when the number of samples is small. This algorithm is based on calculating the distances (e.g., Euclidean distance) from each object to the rest of the samples within a training set to select the k nearest neighbors. This kNN algorithm enables classifying new objects, considering that an increased k value reduces the impact of errors whereas its decrease worsens classification.

#### 3.3.5. Support Vector Machine

The support vector machine (SVM) is a non-parametric machine learning tool that can be applied for both classification and regression purposes [35]. This technique is based on computing a decision boundary (optimal hyperplane) able to distinguish groups by maximizing the separation between classes. The maximum margin is defined as the double minimum distance from support vector points to the hyperplane. To train SVMs, a supervised learning tool is needed, which is based on an iterative algorithm aimed at minimizing the error of the output. In turn, SVM models can be categorized into two groups according to the form of the error function: SVM type 1 and type 2, also called C-SVM and ν-SVM, respectively. The parameters C (capacity constant) and ν can be estimated by cross-validation algorithms to avoid overtraining, and thereby control the model complexity. Moreover, SVM is also useful in non-linear modeling, when the best separation between classes cannot be achieved with a hyperplane. In that situation, different mathematical functions (i.e., kernel functions) can be used to accomplish the linear discrimination of the original data by means of their projection in a new higher-dimensional space. Here, the Gaussian and polynomial functions are the most commonly employed algorithms, which enable finding a linear solution to a non-linear dataset. Accordingly, an efficient SVM modeling requires carefully optimizing various parameters, including the training constants (i.e., C or ν) and kernel parameters (i.e., γ, which controls the shape of the separating hyperplane), to obtain optimal SVM models able to classify new samples.

#### 3.3.6. Chemometric Approaches Based on Decision Trees

Decision trees are powerful machine learning algorithms based on the use of a tree-like model of decisions and their possible consequences, which can be applied for both classification and regression tasks [36]. Some of the most common decision tree-based techniques include classification and regression tree (CART) analysis, chi-square automatic interaction detection (CHAID), and random forest (RF). CART analysis can be used to predict outcomes based on predictor variables. This algorithm relies on the application of automatic stepwise variable selection to calculate the importance rank of the variables. In this decision tree, nodes are split into sub-nodes on the basis of a threshold value of an attribute. This iterative splitting is repeated until no further desirable sub-nodes are available. Similarly, CHAID also enables discovering relationships between a categorical response variable and categorical predictor variables. To this end, the chi-square test of independence is employed with the aim of identifying significant independent variables in the dataset. Furthermore, the Bonferroni correction is applied for significance adjustment.

In recent years, modern variations of these classical approaches based on decision trees have been developed, RF being the most widely employed. RF is a non-parametric and non-linear tool that relies on a learning strategy known as ensemble learning, which is based on generating decision trees by combining individual trees that are determined by the values of a random vector sampled independently and with the same distributions for all the trees in the forest [37]. These trees are divided into numerous nodes using subsets of randomly selected input variables (m). Accordingly, the most important parameters to be optimized during RF modelling are the value of m and the number of decision trees. The optimum value of these model parameters can be found by k-fold cross-validation as a balance between model complexity and fitness. However, it is well-known that the m value is not critical, so the square root of the total number of attributes is routinely used as the default value in practice. Moreover, the model quality is evaluated by the accuracy rates in the training and testing sets and/or by out-of-bag (OOB) error, which is the prediction error computed from the prediction of the original data that is not employed to build the classification trees. In practice, other measurements such as the Gini index can be applied for assessing the quality of a particular node, since this index can be regarded as a measure of node purity (i.e., a small value indicates that a node is mainly composed of objects from a unique class). The Gini index can also be employed for evaluating the discriminant capacity of each variable, i.e., the relevance of each feature in the model. Thus, the mean decrease of the Gini index (MDGI) is useful for selecting the most important variables for inter-class discrimination.

#### 3.3.7. Artificial Neural Networks

An artificial neural network (ANN) is a nonlinear pattern recognition technique based on imitation of the functioning of biological nervous systems, which is capable of predicting categorical and quantitative variables [38]. In general, an ANN is made up of neurons that are organized in different layers (one input layer, one or more hidden layers, and one output layer), with unidirectional connections from input to output. These synaptic connections (the connections to and from neurons) indicate the flow of the signal through the network and are associated with the corresponding synaptic weight. To imitate the functioning of the neural networks of living organisms, the artificial neural network uses two algorithms, one for calculating the weighted sum of the input values (predictor variables) and another called the activation function that generates the response or output, which in classificatory analysis would be the probability that a sample belongs to each of the categories. Through an iterative trial-and-error procedure, the parameters that characterize the neural network are established, such as the number of neurons, hidden layers, their arrangement or architecture, synaptic weights, etc. The most common ANN approach is the multi-layer perceptron (MLP), successfully used for classification and pattern recognition. ANNs need data learning in order to be trained with the aim of functioning properly. For this, the links between inputs and outputs are first identified in a training set and a test set is then employed to evaluate the prediction ability. ANN training is normally achieved by back propagation, a method aimed at reducing the error committed by the neural network on the training step. Once the ANN algorithm learns the idiosyncrasies of the training set, the model is prone to overfitting. To avoid this, a third sample set (validation set) is needed to estimate the generalization ability in order to make accurate predictions of new objects. The weights of the hidden layers can be adjusted to minimize the training error; this optimization is finished when the validation error begins to rise. The efficacy of the training step can be evaluated by the root-mean-squared (RMS) errors.

### 3.4. Regression and Prediction

Multivariate calibration techniques, based on statistical regression and prediction, are aimed at determining relationships between response and independent variables [39]. To this end, these methods are based on estimating efficient mathematical models able to interpret the performance of the system and to accurately predict the characteristics of future samples.

#### 3.4.1. Multiple Linear Regression

Multiple linear regression (MLR) is commonly employed for calibration and regression in chemistry. The association between the response and the input variables is measured by standardized regression coefficients that are obtained by the method of least squares. The application of MLR requires the following conditions: dependent and independent variables must be linearly inter-related, the observations must be independent, normality and homoscedasticity of the residuals, absence of influential points (i.e., outliers), and no multicollinearity. Multicollinearity occurs when the explanatory variables are highly intercorrelated, which may consequently provoke imprecision or instability during the estimation of the model parameters, thereby affecting the accuracy of the model prediction. This can be evaluated by computing the correlation between pairs of independent variables or by calculating the variance inflation factor (VIF). The VIF parameter is indicative of the variance increase of an estimated regression coefficient with respect to the ideal situation where the explanatory variables are strictly independent. Therefore, a high VIF value denotes the presence of multicollinearity, being usually employed a threshold equal to 5 or 10, depending on the domain. A simpler way to minimize the VIF parameter is to discard highly intercorrelated variables or, eventually, to standardize the data. To confirm the statistical validity of the model, the coefficient of determination (R^2^), an estimate of the proportion of the global variation in the data that is explained by the model, is used as an indicator of the goodness of fit.

#### 3.4.2. Principal Component Regression

Principal component regression (PCR), based on classical PCA, applies linear regression modeling to predict the outcome on the basis of the covariates, for which the PCs of the explanatory variables are employed as regressors. Accordingly, PCR transforms a set of correlated variables into a set of uncorrelated PCs. Therefore, one major application of PCR is to solve multicollinearity issues. PCR overcomes the collinearity problem by excluding low-variance PCs in the regression step.

#### 3.4.3. Partial Least Squares Regression

This is a widely used multivariate statistical technique whose objective is to find latent variables by projecting the X and Y variables in a new space where their covariance is maximized. A PLS model will try to find the multidimensional direction in the X space able to explain the maximum variance direction in the Y matrix. PLS regression is of particular utility in avoiding overfitting when the number of explanatory variables is large and they are intercorrelated. This is because during PLS modeling most of the variance is explained by the first latent variables, whilst the remaining variance mostly describes random noise or linear relationships between dependent and independent variables. The optimal number of components is usually obtained using cross-validation tools by calculating a statistic for lack of prediction accuracy called prediction residual sum of squares (PRESS). Thus, the optimum number of components will be the one that produces the lowest PRESS value. Furthermore, their predictive ability can also be evaluated by calculating the regression correlation coefficient (R^2^) and the residual predictive deviation (RPD).

#### 3.4.4. Orthogonal Partial Least Squares Regression

Orthogonal partial least squares regression (OPLS) is a supervised method that is commonly employed to find the relationship between a set of predictor variables (X) and one or more responses (Y). Basically, OPLS is intended to extract the maximum information that reflects the variation in the original dataset, while assuming the existence of a small subset of hidden variables able to predict the response variables. These subsets are formally known as latent variables. The OPLS method uses orthogonal signal correction to maximize the explained covariance between X and Y on the first latent variable, while the remaining components capture variance in X orthogonal to Y.

## 4. Application of Chemometrics to Olive Oil Authenticity Research

To contextualize the above-provided overview on the application of chemometrics in food authenticity and traceability research, in this section we review some recent literature considering olive oil as an example. 

Using an LC-MS platform, Becerra-Herrera et al. characterized the phenolic profile of extra-virgin olive oil (EVOO) samples and then applied various chemometric approaches to discover chemical descriptors to differentiate between different PDOs [40]. An exploratory PCA revealed that only eight of the variables under investigation accounted for most of the variability in the PCs that were obtained, namely tyrosol, hydroxytyrosol, p-HPEA-EDA, 3,4-DHPEA-EDA, 3,4-DHPEA-EA, pinoresinol, secoiridoids, and total phenolic compounds. Afterward, LDA modeling enabled the accurate classification of EVOOs according to their geographical origin (cumulative variance = 93.4%). In another study, traditional chromatographic methods aimed at characterizing the unsaponifiable fraction were combined with NMR fingerprinting to investigate the influence of variety and geographical origin in the chemical composition of olive oils collected from different Andalusian locations [17]. Then, complementary supervised pattern recognition procedures (i.e., LDA, PLS-DA, SIMCA) and machine learning algorithms (i.e., RF, ANN, SVM) were employed to build classification and predictive models. The most discriminant variables between the study groups were found to be tocopherols, squalene, a few sterols (campesterol, stigmasterol, β-sitosterol), aliphatic alcohols (heptacosanol, octacosanol), and NMR signals associated to fatty acid chains. Furthermore, the statistical models that were built with these data provided a clear discrimination according to the variety and to a lesser extent the geographical origin.

Multi-elemental analysis, in combination with advanced chemometric techniques, has also demonstrated great utility in evaluating the geographical origin of Spanish EVOO samples [19]. Preliminary modelling using PCA and LDA showed that the samples under investigation could be grouped into three major clusters: Atlantic coast (Huelva province), Mediterranean coast, and inland regions. Then, various multivariate models (i.e., PLS-DA, SVMs, RF) were developed and validated using minerals as candidate markers. The results evidenced that EVOO samples collected in the province of Huelva have a distinctive multi-elemental profile because of the characteristic geochemistry of this area. On the other hand, EVOOs produced in the Mediterranean and inland regions were more similar, only slight differences being detected in terms of iron and titanium. 

As a complement to conventional chromatographic profiling approaches, metabolomics stands out as a very powerful tool to comprehensively characterize the phytochemical profile of olive oil. In this vein, Senizza et al. recently applied untargeted LC-MS-based metabolomics to 408 Italian EVOO samples, corresponding to different varieties, origins, and blends [41]. Multivariate discriminant analysis by means of OPLS-DA enabled identifying specific markers of authenticity, cholesterol derivatives and phenolic compounds (tyrosols, oleuropeins, stilbenes, lignans, phenolic acids, and flavonoids) being the best discriminant variables. Finally, ANN modelling provided a satisfactory performance in discriminating authentic Taggiasca samples (sensitivity = 100%). On the other hand, other non-targeted fingerprinting approaches based on spectroscopy have also been proven to represent a suitable analytical strategy in olive oil research. For instance, González-Domínguez et al. employed a rapid luminescent method to characterize edible oils and detect adulterations [25]. In this study, a regression model was successfully built on the basis of five luminescent frequencies related to minor oil components. Interestingly, this model provided excellent performance in the detection of virgin olive oil adulterated with hazelnut oil. 

## 5. Conclusions

Multiple factors (e.g., geographical origin, variety/breed, manufacturing practices) may influence the chemical composition of foods and therefore have a considerable impact on their organoleptic, nutritional, and bioactive properties. For this reason, reliable, cost-effective, and powerful analytical methods are needed to ensure the authenticity and traceability of food products. However, the importance of properly addressing data processing and analysis steps is often underestimated in this field. In this respect, the use of advanced chemometric tools is mandatory in building multivariate models for classification, discrimination, and prediction, and for identifying candidate authenticity and traceability markers. 

The choice of the most appropriate statistical tool to apply depends on several factors related to the nature of the dataset, including the definition of the class criteria, the homogeneous distribution of the sample, the number of input variables, and the number of samples. However, it is common practice to apply various classification techniques and evaluate their fitness for the problem under study [42]. This is usually accomplished by combining unsupervised pattern recognition methods for a first exploration of the data, with subsequent application of supervised learning approaches [43]. In this respect, PCA and HCA stand out as the most commonly employed unsupervised multivariate tools, whereas LDA and PLS-DA have demonstrated suitable performance for discrimination purposes in food science. Nevertheless, the use of state-of-the-art machine learning tools (e.g., RF, SVM, ANN) has rapidly emerged in recent years. Regardless of the multivariate approach, another crucial factor to be considered is the proper preprocessing of the data matrix, with the aim of enhancing data quality and removing unwanted variations prior to statistical analysis. Furthermore, after statistical modelling, the implementation of reliable validation tools is of utmost importance in evaluating model performance and checking the absence of overfitting.

## Figures and Tables

**Table 1:** Summary of analytical approaches commonly employed in food authenticity and traceability.

Analytical Approach	Advantages
Chromatographic methods	Quantitative and qualitative analysis Versatility Accuracy and precision
Multi-elemental methods	Geographical origin (e.g., rare earths) Production conditions (e.g., isotopic ratios)
Metabolomics methods	Wide coverage High-throughput analysis
Spectroscopic methods	Simplicity and rapidity High-throughput analysis Non-destructive nature Low cost
Molecular biology methods	Specificity and sensitivity

## Data Availability

No new data were created or analyzed in this study. Data sharing is not applicable to this article.
